# Straightforward Synthetic Protocol to Bio-Based Unsaturated Poly(ester amide)s from Itaconic Acid with Thixotropic Behavior

**DOI:** 10.3390/polym12040980

**Published:** 2020-04-22

**Authors:** Lazaros Papadopoulos, Marcel Kluge, Dimitrios N. Bikiaris, Tobias Robert

**Affiliations:** 1 Laboratory of Polymer Chemistry and Technology, Department of Chemistry, Aristotle University of Thessaloniki, GR-541 24 Thessaloniki, Greece; lazaros.geo.papadopoulos@gmail.com (L.P.); dbic@chem.auth.gr (D.N.B.); 2 Fraunhofer Institute for Wood Research—Wilhelm-Klauditz-Institut WKI, Bienroder Weg 54E, 38108 Braunschweig, Germany; marcel.kluge@wki.fraunhofer.de; 3 Institute for Chemistry of Renewable Resources, Department of Chemistry, University of Natural Resources and Life Sciences Vienna, Konrad-Lorenz-Straße 24, 3430 Tulln, Austria

**Keywords:** bio-based polymer, poly(ester amide), itaconic acid, thixotropy, photo-DSC

## Abstract

In the field of polymer chemistry, tremendous efforts have been made over the last decade to replace petrochemical monomers with building blocks from renewable resources. In this respect, itaconic acid has been used as an alternative to acrylic acid or maleic acid in unsaturated polyesters for thermal or UV-curing applications. However, examples of poly(ester amide)s from itaconic acid are scarce. Under standard polycondensation reactions, the presence of free amines leads to aza-Michael addition reactions at the α,β-unsaturated double bond of the itaconic acid and isomerization reactions to mesaconic acid. Both reactions make the resulting materials useless as UV-curing polymer resins. To avoid these undesired side reactions, we herein report the use of preformed, well-defined diols containing internal amide bonds. The resulting unsaturated poly(ester amide) resins were analyzed before and after UV-induced crosslinking. Viscosity measurements revealed a strong thixotropic behavior induced by the amide groups, which is usually not detected in structurally similar polyester resins.

## 1. Introduction

Over the last years, polymers from renewable resources have attracted considerable attention from both industry and academia in an effort to limit the environmental impact arising from the excessive use of polymeric materials derived from fossil resources [1,2,3,4]. However, in order to be able to compete with these established materials, the bio-based counterparts have to be either economically competitive or exhibit unprecedented material properties [5,6]. A viable way to achieve the latter is the utilization of bio-based monomeric building blocks, which are not economically accessible from petrochemical pathways. These can be used to produce novel polymeric structures that in turn can lead to materials with new properties [7,8,9].

In this context, itaconic acid (IA) has lately drawn considerable attention as it exhibits a promising structure and is available at competitive prices (<2 €/kg) [10]. This monomer is already produced on industrial scale (>80.000 t/a) via the fermentation of sugars with the fungi *aspergillus terreus* [11,12,13]. Another interesting feature of IA is its versatility. Since it possesses two carboxylic groups and an *exo*-double bond, it is a good substrate for different chemical transformations. This versatility also applies for the production of polymers. Two main strategies emerge: first, the utilization of the unsaturated double bonds for the radical polymerization of homo-polymers and co-polymers [14,15,16,17,18] and second, the synthesis of unsaturated polyesters [19,20,21,22,23,24,25]. Those materials find a number of applications in printing inks, shape memory polymers, and coatings [26,27,28,29,30], while they can also be exploited for post-polymerization reactions [31,32,33,34].

Despite the increased research activities in itaconic acid-based materials described above, poly(ester amide)s from itaconic acid have so far received little attention. The main reason for this is the fact that the α,β-unsaturated double bond is prone to a nucleophilic attack of the diamine, leading to the formation of pyrrolidone rings [35,36]. While this effect can be exploited for the synthesis of bio-based polyamides and poly(ester amide)s [37,38], it also results in the complete conversion of the unsaturated double bonds and thus the inability to produce polymers susceptible to radical curing reactions. Recently, we reported two synthetic strategies in order to circumvent these undesired side reactions [39]. The first method involved the synthesis of an itaconic acid-based oligoester and an oligo(ester amide), both of which were synthesized independently. In the next step, these two oligomers were combined in a transesterification reaction. The second synthetic pathway involved the in situ formation of a bis-ester amide that was subsequently reacted with IA and further monomers. While both approaches resulted in the successful synthesis of the envisaged structures, the first synthetic strategy involved three synthetic steps, which makes the synthesis quite cumbersome. The second strategy suffered from a base-catalyzed isomerization of the itaconate moiety to the mesaconate, which was due to some free amines still present after the in situ formation of the bis-ester amide.

Another promising and more straightforward approach to this class of unsaturated poly(ester amide)s is the used of well-defined preformed monomers containing amide moieties. In this respect, we recently reported the use of symmetric diamido-α,ω-diols (amido diols) as building blocks for poly(ester amide)s derived from succinic acid [40,41,42]. By using these monomers, we were able to prevent the formation of succinimide that usually interferes with the synthesis of polyamides or poly(ester amide)s derived from succinic acid. Intrigued by these promising results, we were interested in whether these amido diols could also be used as building blocks for poly(ester amide)s derived from itaconic acid. This would allow for the introduction of amide segments without the occurrence of the undesired aza-Michael additions described above. In this work, two series of unsaturated poly(ester amide)s were synthesized based on itaconic acid and two different preformed amido diols. The properties of the polymer resins as well as UV-cured materials were thoroughly characterized, revealing a thixotropic behavior of the resins and considerable influence of the amide content on the thermal and mechanical properties of the final cured materials.

## 2. Materials and Methods

### 2.1. Materials

Dimethyl itaconate (DMI, 98%), dimethyl sebacate (DMS, 98%), 1,4-diaminobutane (≥98%), and 1,6-diaminohexane (≥99%) were purchased from Tokyo Chemicals Industry (TCI), Tokyo, Japan. Glycerol (Gly, ≥98%) and chloroform-*d*_1_ (99.8% D) + 0.03% TMS *v*/*v* were provided by Carl Roth, Karlsruhe, Germany. 2,3-Butanediol (2,3-BD, a mixture of meso-D- and L-form, ≥99%) and 2,6-di-*tert*-butyl-4-methylphenol (BHT, 99%) were bought from Merck, Darmstadt, Germany. 4-Methoxyphenol (MeHQ, 99%) was purchased from Sigma-Aldrich Chemie, Steinheim, Germany. ε-Caprolactone (99%) was obtained from Alfa Aesar, Kandel, Germany. 1,3-Propanediol (1,3-PD, 99.7%) was kindly provided by DuPont Tate & Lyle Bio Products, Loudon, NH, USA. Isosorbide (Is) was obtained from Ecogreen Oleochemicals, Dessau-Roßlau, Germany. FASCAT 4101 catalyst was provided by PMC Group, Mount Laurel, NJ, USA. Solvents were reagent or analytical grade and were purchased from VWR International, Fontenay-sous-Bois, France. All reagents were used without further purification except for isosorbide, which was recrystallized from ethyl acetate.

### 2.2. Measurements

FTIR spectra were obtained on a Thermo Scientific Nicolet iS5 FTIR (Thermo Fisher Scientific, Waltham, MA, USA) using the ATR technique (32 scans, resolution of 4 cm^−1^).

NMR experiments were conducted on a Bruker Avance III 400-MHz spectrometer (Bruker, Billerica, MA, USA). ^1^H NMR shifts of polymers are reported in ppm (δ) downfield from tetramethylsilane (TMS) and were determined by reference to the residual solvent peak (chlorofrom-*d*_1_, 7.26 ppm for hydrogen atoms).

Size-exclusion chromatography (SEC) was performed on a Malvern Viscotek GPCmax (Malvern Instruments, Malvern, United Kingdom) equipped with triple detection, consisting of a Malvern Dual detector and Schambeck RI2012 refractive index detector. The separation was performed by utilizing two PLgel 5 mm MIXED-C, 300 mm columns from Agilent Technologies (Santa Clara, CA, USA) at 35 °C. Chloroform was used as the eluent at a flow rate of 0.5 mL min^−1^. Data acquisition and calculations were performed using Viscotek OmniSec software version 5.0. The samples were filtered over a 0.2 mm PTFE filter prior to injection.

Rheological properties of resins were studied on a Bohlin CVO 100 rheometer (Bohlin Instruments, Cirencester, UK) equipped with cone-plate geometry (CP 4°, 40 mm). For the table temperature scan, measurements were done with a shear rate of 100 s^−1^ for 10 s. Five measurements were carried out for each temperature (25 °C, 37.5 °C, 50 °C, 62.5 °C, and 75 °C), and the average value was calculated for each temperature. For the shear thinning tests and to measure time-dependent behavior, each sample was left on the rheometer for five minutes prior to measurement to ensure a temperature equilibrium of 25 °C. Subsequently, a shear rate of 1 s^−1^ was applied for three minutes in order to reach the equilibrium state. Then, the shear rate was increased at 100 s^−1^ for the application of the mechanical load for a duration of two minutes. Finally, a shear rate of 1 s^−1^ was applied again over a prolonged period of time until the system reached an equilibrium state again. The ability of recovery as a measure of thixotropic behavior for each system was evaluated by comparing the viscosity value of the first and the third stage of the measurement. Five samples of each resin were studied, and the average values are given in the results.

Photo-differential scanning calorimatry (Photo-DSC) measurements were conducted on a DSC 3+ (Mettler-Toledo, Greifensee, Switzerland) equipped with a Lightningcure LC8 UV spot light source (Hamamatsu Photonics, Hamamatsu, Japan) at 70% of its intensity. To get the integration of the heat of reaction, two runs were carried out with a short break between the runs to let the resin cool down. The second run was made once the material was fully cured and the baseline was stable. Then, the second curve was subtracted from the first to obtain the curve related to the curing only. Each run is conducted as follows: 30 s at the set temperature (25 °C at atmospheric pressure under air) without the lamp; then, the lamp is turned on for 2.5 min. The break between the runs lasts 30 s.

Dynamic mechanical analysis (DMA) was carried out on a Tritec 2000 DMA (Triton Technology, Loughborough, UK) in dual cantilever bending mode. Measurements were performed on rectangle bars (80 × 10 × 2 mm) which were prepared using a Teflon mold. Irgacure-1173 was added to the resin as photoinitator (5 wt %). Before curing, the mixture was left for 1 h at elevated temperature (50 to 90 °C depending on the viscosity of each sample) to reduce the amount of air trapped in the resin that could lead to cracks. Subsequently, it was poured into the mold and cured under UV light using an Aktiprint mini UN50029 UV dryer (Technigraf, Grävenwiesbach, Germany), equipped with a UV 4/120-2 lamp (254 nm, max. 120W cm^−1^). The conveyor belt was set at 5 m min^−1^, and five runs were required to fully cure the specimens. The irradiation energy (UV dose) was measured before and after curing with an average value of 460 mJ cm^−2^. Samples were studied under nitrogen in a temperature range of −30 to 120 °C (5 K min^−1^, 1 Hz, maximum displacement 0.05 mm). The exact dimensions of specimens were determined before each experiment, and five test bars were measured for each sample.

Thermogravimetric analysis (TGA) of cured resins was performed on a TGA/DSC 1 (Mettler-Toledo, Greifensee, Switzerland) under nitrogen (35 mL min^−1^). Samples of ca. 10 mg were heated from 25 to 800 °C with a heating rate of 10 K min^−1^.

### 2.3. Synthesis of Amido Diols

Two amido diols (AD) with varying alkylene chain lengths were synthesized as described previously [40,42]. In brief, a certain amount of diamine (1,4-diaminobutane or 1,6-diaminohexane) was placed in a three-necked flask equipped with mechanical stirrer and cooler and dissolved in 2-propanol (200 mL mol^−1^). ε-Caprolactone (2 equivalents) in 2-propanol (50 mL mol^−1^) was slowly added to the diamine solution at room temperature using a pump (0.6 mL/min). Stirring was continued overnight. The resulting white pasty product was diluted with acetone and then washed several times with the same solvent to remove residual monomers and potentially formed oligomeric by-products by (homo)polymerization of the lactone. It was further purified by recrystallization from methanol and acetone and then dried under reduced pressure, whereby the respective product was obtained as a white powder. Amido diols are named analogously to polyamides of the AABB type, while the first number indicates the number of carbon atoms in the diamine and the second indicates the number of carbon atoms in the lactone. Hence, AD 4.6 was obtained from 1,4-diaminobutane and ε-caprolactone, while AD 6.6 was synthesized from 1,6-diaminohexane.

### 2.4. Polymer Synthesis

Six unsaturated poly(ester amide) (PEA) resins as well as a neat polyester, that served as a reference, were prepared via the transesterification method, where an excess of 30% hydroxyl groups was targeted (dimethyl ester:diol ratio of 1:1.3). As an example, the synthesis of the neat polyester resin, named PE, will be described. For the synthesis of 60 g of resin, 35.57 g of DMI (224 mmol, 0.8 eq), 12.95 g of DMS (56 mmol, 0.2 eq), 14.71 g of 1,3-PD (193 mmol, 0.75 eq), 5.14 g of Is (35 mmol, 0.125 eq), 3.24 g of Gly (35 mmol, 0.125 eq) and 6.33 g of 2,3-BD (70 mmol, 0.25 eq), along with 0.12 g of FASCAT 4101 (0.2 wt %), 24 mg of BHT (0.08 wt %), and 18 mg of MeHQ (0.06 wt %) were charged into a three-necked round-bottom flask equipped with mechanical stirrer, thermometer, condenser and an immersed thermocouple to control the temperature of the heating mantle. The mixture was heated gradually until homogenization, and then the temperature was set at 180 °C for 3 h. Finally, a slight vacuum (20 mbar) was applied for 30 min, and then the system was left to cool down to room temperature. The product was obtained as a yellow-orange resin. For the preparation of the resins containing amide moieties in their backbone, 0.0625, 0.125, and 0.1875 eq of 1,3-PD were substituted by the respective amido diol.

## 3. Results and Discussion

### 3.1. Polymer Synthesis

One of the main objects of this work was the introduction of amide moieties into the backbone of a bio-based unsaturated polyester resin derived from itaconic acid. As it was already discussed, the presence of free –NH_2_ moieties leads to the formation of the pyrrolidone lactam ring from itaconic acid, while it also supports the isomerization of itaconic acid to mesaconic acid. For this reason, preformed symmetrical diols that have two internal amide bonds were used for the synthesis of poly(ester amide)s. In order to assure that the amido diol structure would remain intact under the conditions applied for the synthesis, the dimethyl esters of the respective acids were used. Previous studies have shown that the amido diol structure can be lost by an acidolysis-type reaction if free carboxylic acid groups are present during reaction [43,44,45]. Hence, the dimethyl esters of dicarboxylic acids were utilized to avoid this undesirable side reaction.

Another challenge in the synthesis of poly(ester amide) resins is the formation of hydrogen bonds, which should lead to a high increase in viscosity. Indeed, preliminary studies showed very high viscosity values for the produced resins, rendering them nearly non-processable. In an attempt to counter this effect, a range of bio-based diols were incorporated in the polymer backbone to obtain highly irregular polymer chains, which should reduce the intermolecular interaction. Therefore, we anticipated obtaining poly(ester amide) resins with low viscosities. Even though this strategy was successful to some extent, the phenomenon could only be mildly suppressed, as the viscosity of the resins increased dramatically even with very small amido diol content. So, in another attempt to reduce the viscosity, the reaction time was set at 3.5 h for all resins, as prolonged reaction times can lead to undesired side reactions and an increased molecular weight that would result in higher viscosities of the resins.

**Scheme 1:**
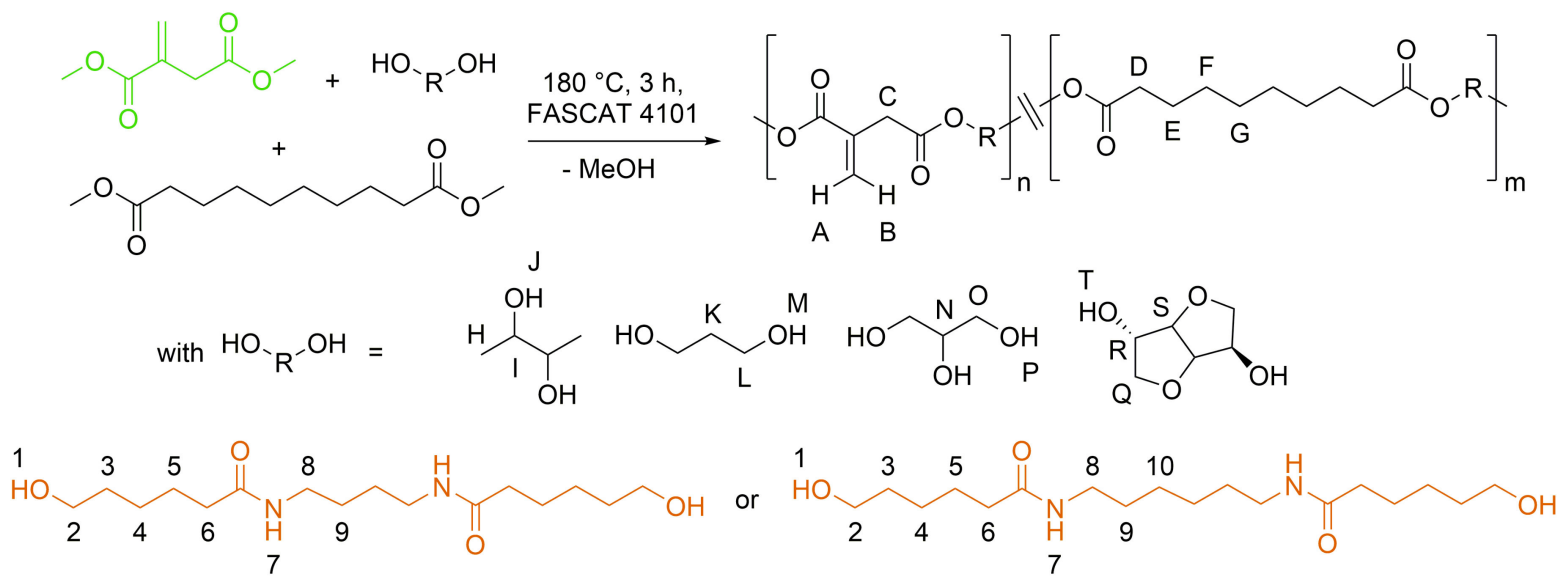
Synthesis route of the unsaturated polyester and poly(ester amide) resins obtained in the course of this study.

Six unsaturated bio-based poly(ester amide) resins containing 5 to 15 mol% of an amido diol (AD 4.6 or 6.6) and a neat polyester, that served as a reference, were successfully synthesized via a transesterification method of DMI, DMS, and a variety of sterically demanding diols (Scheme 1). The distance between amide bonds was varied by the choice of the diamine used for the synthesis of amido diols. All polymers were obtained following the same protocol, considering the above-mentioned limitations. The monomers and composition used for the synthesis of the resins are given in Table 1. PEA labels include the molar fraction of targeted ester-amide sequences in the resin, which corresponds to the molar percentage of the respective amido diol in the feed. For example, 5 mol% of ester amide moieties were aimed for PEA 4.6-05, corresponding to the equal amount of AD 4.6 in the feed. All building blocks used, but the amido diols, are accessible from renewable resources, resulting in PEA resins with very high bio-based content.

### 3.2. Structural Characterization

**Figure 1:**
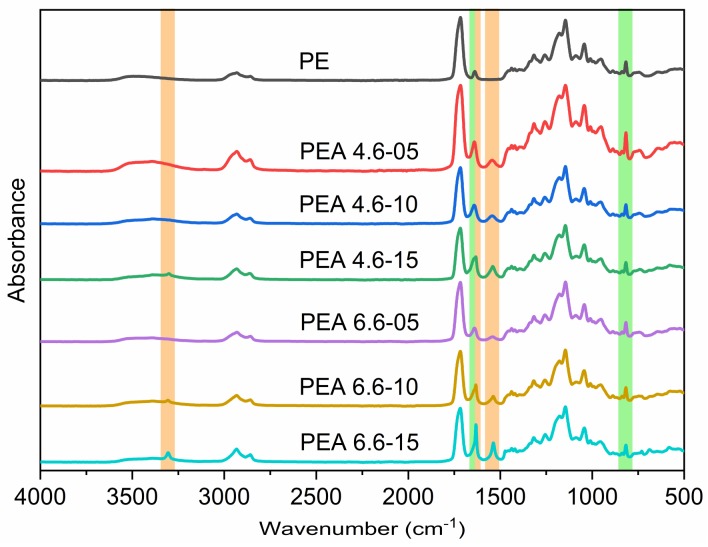
FTIR spectra of synthesized resins. C–C double bond vibrations are highlighted in green, vibrations derived from the amide group are in orange.

The ATR-FTIR spectra of synthesized resins are presented in Figure 1. In the spectra of all polymers, the presence of ester bonds is confirmed by the signal at 1720 cm^−1^, which can be assigned to the C=O stretching vibrations. Peaks at 1640 cm^−1^ and 810 cm^−1^, corresponding to the C–C stretch and C–C deformation vibrations of the double bond of itaconic acid, confirm that the double bond did not undergo undesired side reactions during synthesis. A broad peak around 3500 cm^−1^ can be found in the spectra due to the OH end groups of polymer chains, since diols were used in excess. After the incorporation of an amido diol into the polyester backbone, new signals associated with amide groups can be found in the spectra. The intensity of the signal at 1640 cm^−1^ increased, since the peak of the double bond of itaconic acid and those associated with the C=O (Amide I) stretching vibrations of the amido diol are overlapping. In addition, a new absorption peak can be found at 1540 cm^−1^ that is attributed to the N–H (Amide II) bending vibrations of amide bonds. Furthermore, a new signal is observed at 3300 cm^−1^, which is related to hydrogen-bonded N–H groups. By increasing the amido diol content, the intensity of the peaks associated with amide moieties also increased.

The chemical structure of the synthesized resins was also investigated by means of ^1^H NMR spectroscopy. All the spectra of the resins show the signals of the neat polyester resin (PE). The poly(ester amide)s show additional peaks that can be assigned to the ester-amide sequences. In addition, by increasing the amido diol content, the respective peaks increase in intensity. Detailed analysis of the spectra can be found in the Supplementary Materials.

Both FTIR and NMR spectra support the successful incorporation of the amido diol building block into the polymer backbone. In addition, the results suggest that aforementioned side reactions, such as ring formation or isomerization, can be suppressed when the preformed amido diols are used. Pyrrolidone lactam ring formation was completely avoided, while the isomerization of itaconic acid was below 3%, which is similar to what is observed when standard polyesters are synthesized from itaconic acid. These results confirm that the copolymerization of amido diols is a viable synthetic strategy for the insertion of amide moieties into unsaturated polyester resins based on itaconic acid.

**Figure 2:**
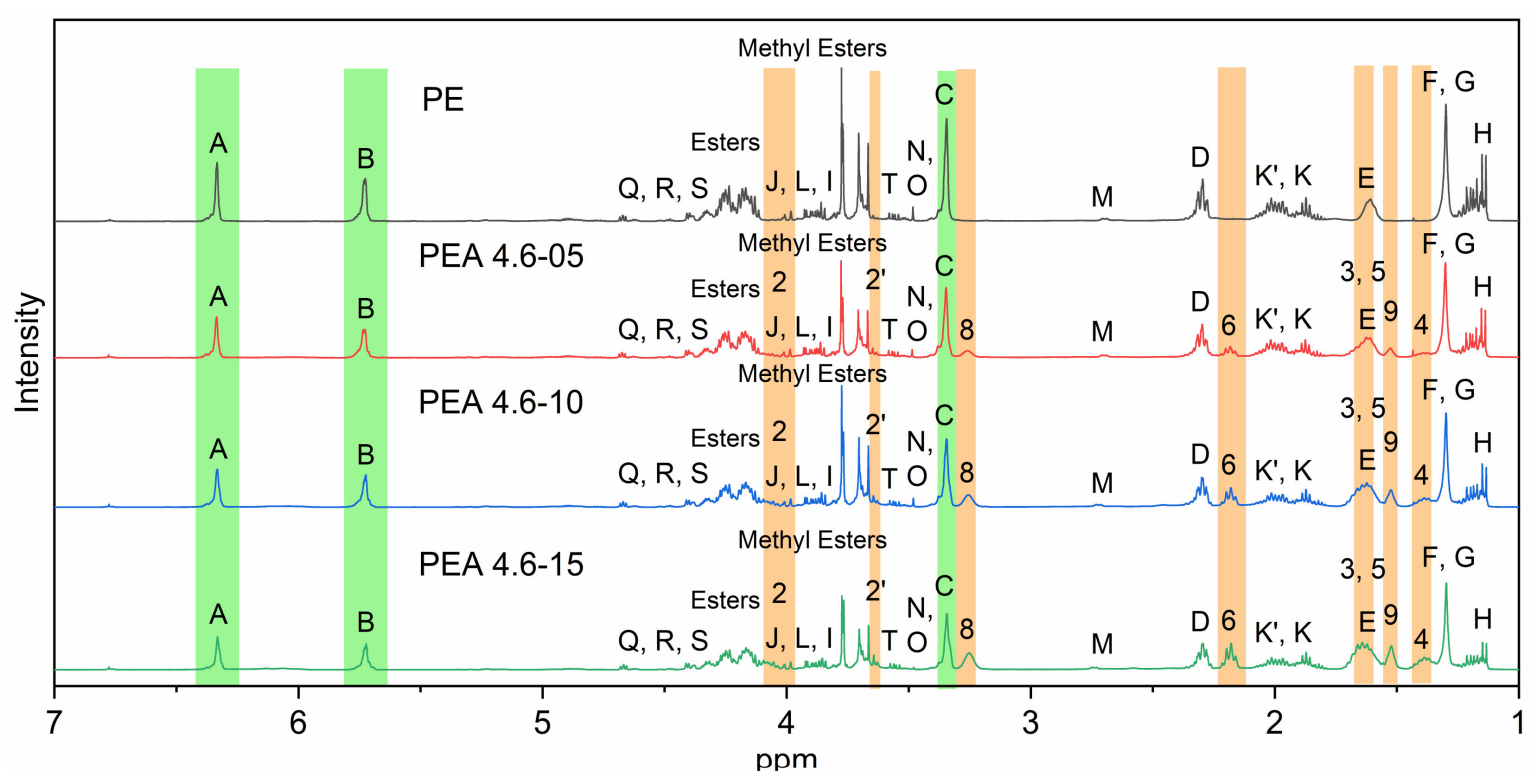
^1^H NMR spectra of the neat polyester resin and PEAs containing different amounts of amido diol 4.6.

As discussed earlier, the reaction time for the synthesis of the resins in question was limited to obtain PEAs with a viscosity suitable for processing. However, this practice resulted in some methyl ester end groups remaining in the polymer chain, as shown in the ^1^H NMR spectra (Figure 2). Therefore, rather low molecular weights are assumed for the resins produced. Nevertheless, we consider this practice mandatory, because the increased viscosity makes these resins very difficult to manipulate for the characterizations needed as well as the envisaged UV-curing applications, and a longer reaction would have rendered them non-processable.

### 3.3. SEC Measurements

**Figure 3:**
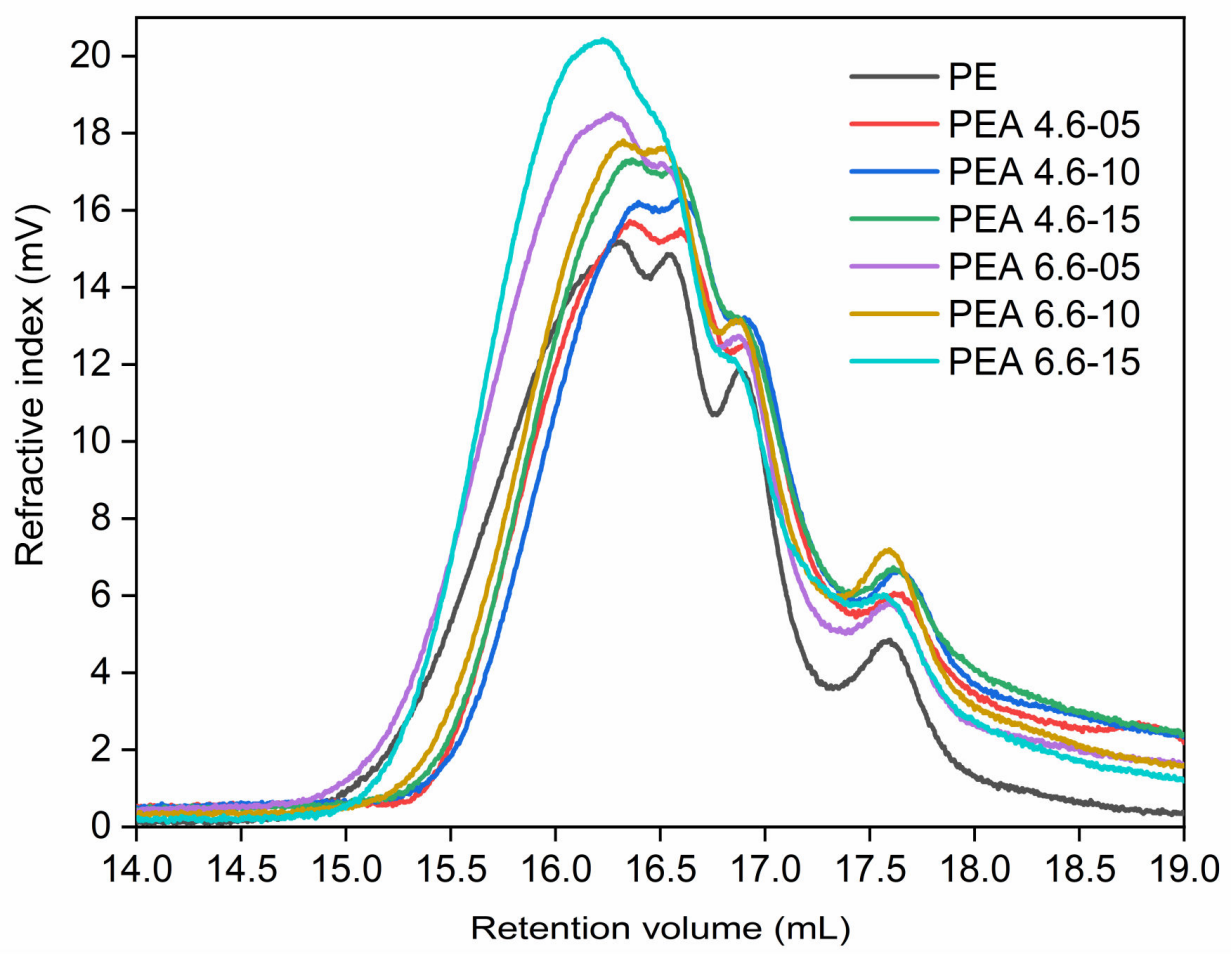
SEC elugrams of the resins synthesized in this study.

SEC results are shown in Figure 3. As expected for the products of polycondensation reactions, the molecular weights of the resins are obtained in gaussian distribution. The absence of any high molecular weight fraction in the elugrams, along with the ^1^H NMR data, shows that undesired side reactions leading to cross-linking of the polymers were successfully prevented. The molecular weight of the produced resins is rather low, as a result of the synthetic procedure, in the range of 300 to 5000 g/mol. However, the M_n_, M_w_, and Đ were not calculated, because the calibration of the measurements was limited to 575 g/mol. Nevertheless, they are still suitable for applications in the coatings field. The resins derived from AD 6.6 present larger retention volume peaks compared to those based on AD 4.6. This indicates that a higher molecular weight is to be expected when the amide groups are separated by a longer alkylene chain.

### 3.4. Viscosity

As anticipated, the incorporation of the amido diol into the polyester backbone led to increased viscosities. However, as a somewhat unexpected feature, the resins also became opaque and almost turned into a waxy solid when allowed to cool overnight in the storage container. At this state, the resins did not flow any more unless an external force was applied. This phenomenon became more pronounced with increasing amido diol content, suggesting that it was derived from intermolecular hydrogen bonds. Similar claims were made in other works where polyesters containing amide moieties were examined, and it was found via temperature-dependent IR spectroscopy that as the temperature increased, the amide-ester hydrogen bonds were destroyed [46,47].

**Figure 4:**
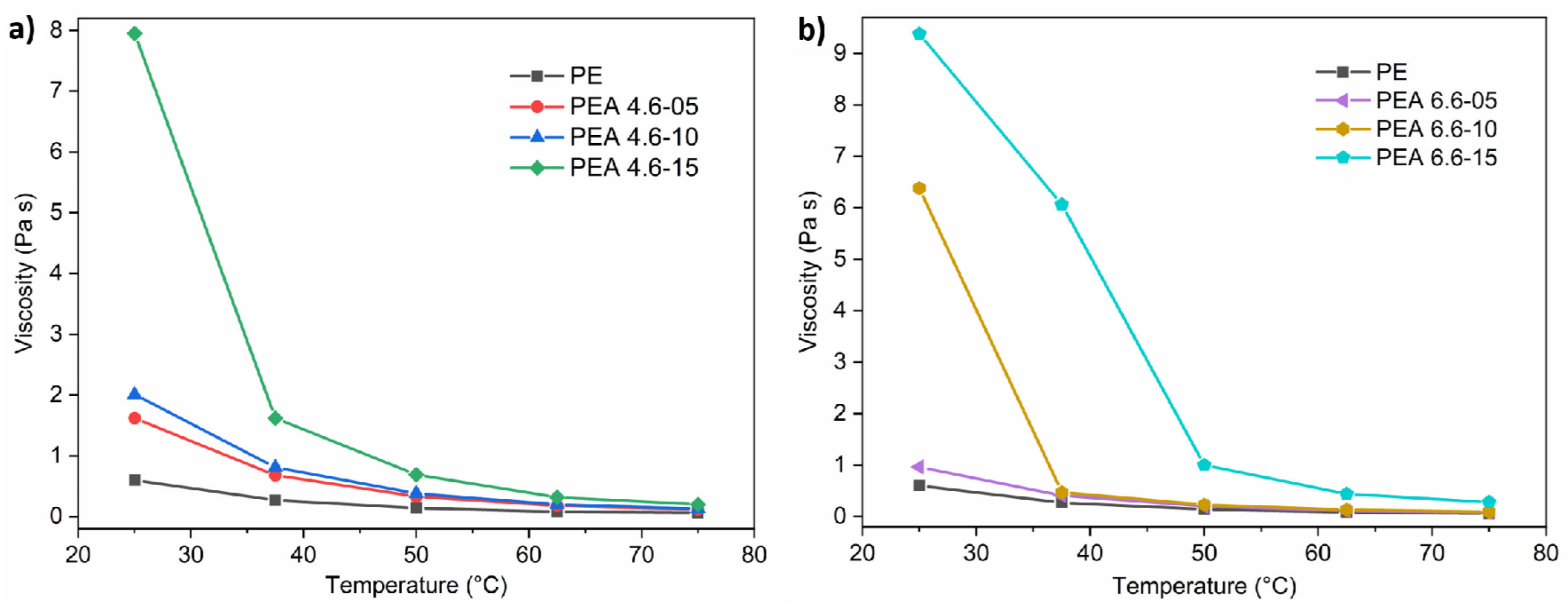
Temperature dependency of the viscosity of resins synthesized in the course of this study with (**a**) amido diol 4.6 and (**b**) amido diol 6.6.

**Figure 5:**
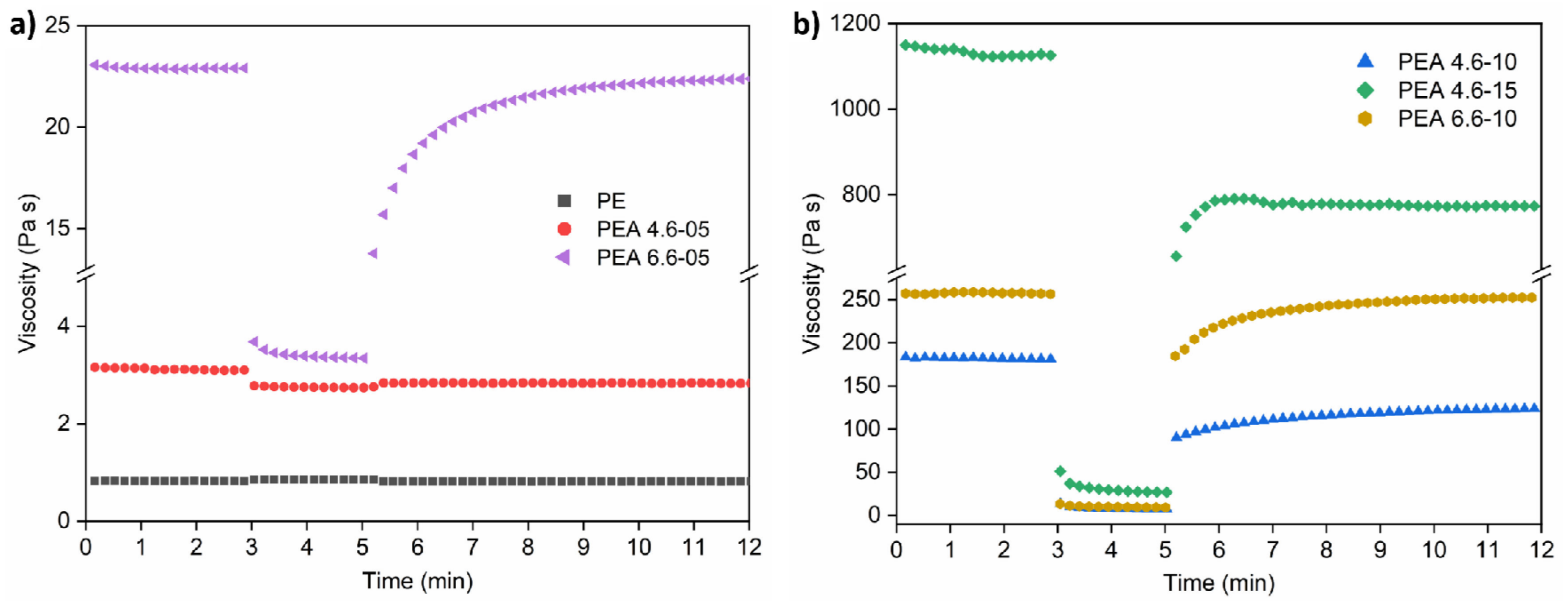
Viscosity experiment conducted to study the thixotropic behavior of the resins containing (**a**) 5 mol% amido diol and (**b**) 10 mol% and higher amounts of amido diol. Conditions: 3 min at 1 s^−1^, then 2 min at 100 s^−1^, finally 7 min at 1 s^−1^.

For the first set of measurements, viscosity values were obtained at different temperatures under a constant shear rate of 100 s^−1^. As can be seen in Figure 4, an increase of the temperature resulted in an exponential decrease of the viscosity. In addition to the temperature dependency, we were intrigued to investigate whether the resins also exhibit a non-Newtonian behavior—thus, if the viscosity can be influenced by a difference in shear stress and whether there is a time-dependent behavior. This behavior was studied by monitoring the viscosity as a function of the shear rate. In detail, this was done by first applying a low shear rate of 1 s^−1^ until a constant viscosity was obtained. Then, a high shear rate of 100 s^−1^ was applied for 2 min to break down the interactions between the polymer chains. In a last phase, the shear rate was reduced again to 1 s^−1^. For both transitions of the shear rates, the effect on the viscosity was examined, as well as a time dependency of the latter, which would indicate a thixotropic behavior. In addition, the ability of the resins to recover to the initial state was examined [48,49,50,51]. A strong shear thinning effect could be observed for all resins with amido diols incorporated in the polymer chain (Figure 5). Even at low amido diol contents of 5 mol%, this effect could be observed with AD 6.6 resulting in a more pronounced influence on the shear thinning behavior (Figure 5b). With increasing ester-amide content, the initial viscosity increased to a point where PEA 6.6–15 could not be measured anymore. However, for PEA 4.6–15, the viscosity is reduced from 1127 Pa·s at 1 s^−1^ to 30 Pa·s at 100 s^−1^, which corresponds to 2.6% of the initial viscosity (Table 2). This shear thinning effect also showed a time-dependent behavior, indicating a thixotropic behavior of the resins. For the neat polyester, no change in viscosity could be observed. The recovery of the initial viscosity was measured in the third phase of the rheology experiment. Here, the thixotropic behavior was more easily observed, as it took several minutes to reach constant viscosities. In addition, it became apparent that resins containing amido diol 6.6 exhibited very high recovery rates that were significantly higher than those of the corresponding resins with amido diol 4.6 as a building block (Table 2). This difference is especially pronounced for the resins with 10 mol% amido diol, where the resin based on AD 4.6 recovers only 69% of the initial viscosity in comparison to 95% in the case of that derived from AD 6.6. These results suggest that in the case of the former amido diol, the initial arrangement of the resin is not achieved within the measurement time, resulting in a lower amount of polymer chain interaction via hydrogen bonds. Most probably, this behavior can be explained by the different structure of the amido diols, since the more flexible structure is the one with higher recovery rates. The increased length of the spacer unit between the two amide bonds (1,6-diaminohexane and 1,4-diaminobutane, respectively) gives the chain an extra flexibility that contributes to the faster recreation of the hydrogen bonds after their structure is destroyed by the high shear rate.

### 3.5. UV-Curing Behavior

**Figure 6:**
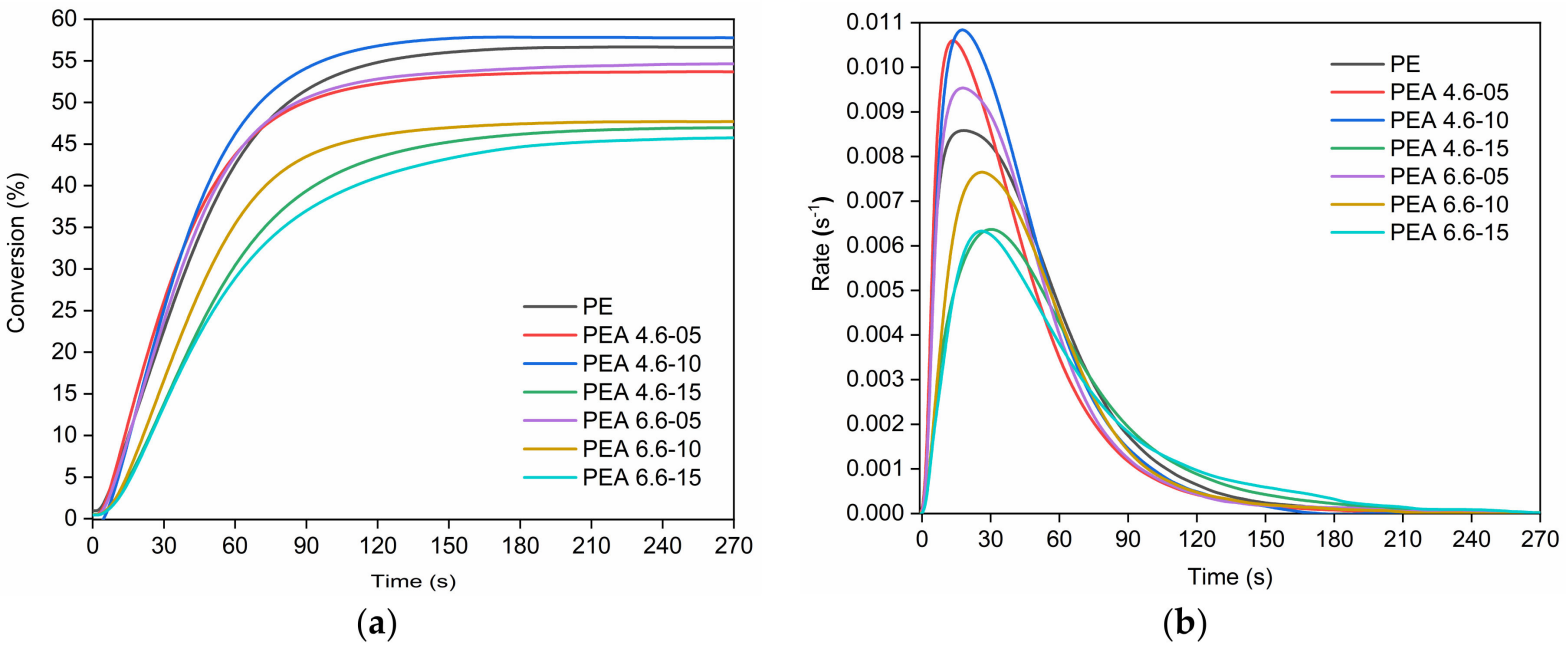
Photo-DSC of the PEAs: Conversion (**a**) and rate of polymerization (**b**).

Photo-DSC measurements were conducted to evaluate the reactivity of the resins toward UV-curing. The double-bond density (DBD) of the resin was calculated from the composition of the resins. In order to obtain the theoretical enthalpy of the UV-induced radical polymerization reaction, the double-bond density was multiplied by the heat of polymerization of dimethyl itaconate (60.67 KJ/mol), which was determined by Dainton et al. [52]. The experimental enthalpy, conversion, and rate of polymerization (ROP) of all resins were calculated from the photo-DSC measurements. The results are all shown in Table 3, and Figure 6 depicts the conversion and ROP over time. The enthalpy, as well as the conversion decreases with increasing ester-amide content. This can be explained by the lower DBD, as well as the increasing viscosity of the resins. The latter compromises the mobility of the polymer chains and therefore the likelihood of radical cross-linking reactions between double bonds. The only exception to the reduction in conversion and enthalpy can be found for PEA 4.6-10, which surpasses the conversion of the neat polyester. The ROP of the polyester is lower than some of the PEAs, but again, a general decreasing trend can be observed with increasing amide content with values between 6.3 and 10.8 × 10^−3^ s^−1^. In general, the values are quite low for UV-curing materials, but they are in the same range that have been reported for other materials derived from itaconic acid [39,53].

### 3.6. DMA Analysis

**Figure 7:**
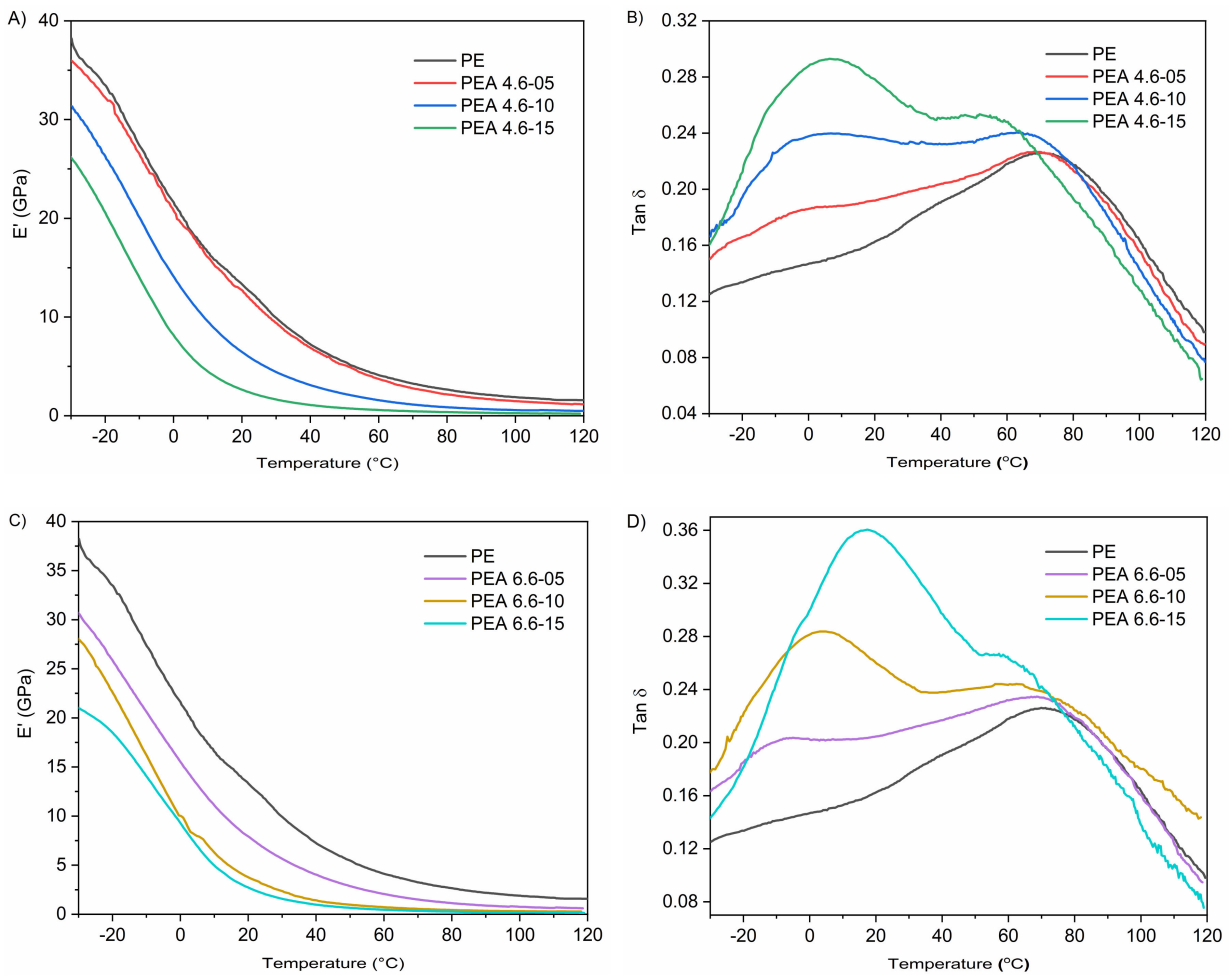
Storage modulus (E’) and tan δ values of the PEAs after UV curing. PEAs with amido diol 4.6 (**A**,**B**) and PEAs with amido diol 6.6 (**C**,**D**).

The PEAs were also cast into a Teflon mold and cured under UV light. The resulting bars (80 × 10 × 2 mm) were analyzed by means of DMA measurements. Figure 7 shows the storage modulus (E’) as a function of the temperature for the crosslinked polymers. The values for all materials at 25 °C are between 11.74 and 2.03 GPa and decrease with increasing temperature, reaching the range of 1.58–0.14 GPa in the rubbery state. The storage modulus E’ is a measurement of material stiffness and can be used as providing the information regarding the molecular weight of a polymer and its crosslink density [54]. By introducing the long aliphatic chain of the amido diols into the structure of the polyesters, the spacing between the double bonds increases, leading to a decrease of the crosslinking density in the cured polyesters. The phenomenon gets more pronounced by the introduction of a higher amido diol content into the resins, as well as by the use of AD 6.6, in which the amide bonds are separated by a longer alkylene chain compared to AD 4.6. This statement is supported by the crosslinking density values (ν_e_) i.e., the molar number of elastically effective network chains per cubic centimeter of a sample, which was calculated from the formula: v_e_ = E’/3RT, where E’ is the storage modulus, R is the gas constant, and T is the Kelvin temperature, which are given in Table 4.

Tan δ graphs are also presented in Figure 7. The tan δ peak corresponds to the T_g_ of the materials, while its broadening occurs when a less homogenous network is created [23,55]. In all cases, a very broad tan δ peak can be observed. The tan δ peak of the cured neat polyester resin is found at 71 °C, and by increasing the amido diol content, a proportional decrease is observed. Simultaneously, the incorporation of the amido diol into the resins resulted in a second peak emerging in the tan δ graphs at temperatures below 0 °C. This peak is shifted to higher values and gains in intensity with increasing AD content in the resins. This is an indication that possibly the crosslinked and non-crosslinked regions of the polymer are affected in different ways from the addition of the amido diols. As the distance between the double bonds is increasing, a more sparsely crosslinked region is created, and this is reflected on the transfer of the peak from 71 °C to lower values. In the same time, movement restrictions of the non-crosslinked polymer chains get lifted, and they can interact more with each other, resulting in more hydrogen bonds being formed and thus the second peak shifting to higher temperatures.

### 3.7. Thermal Degradation

**Figure 8:**
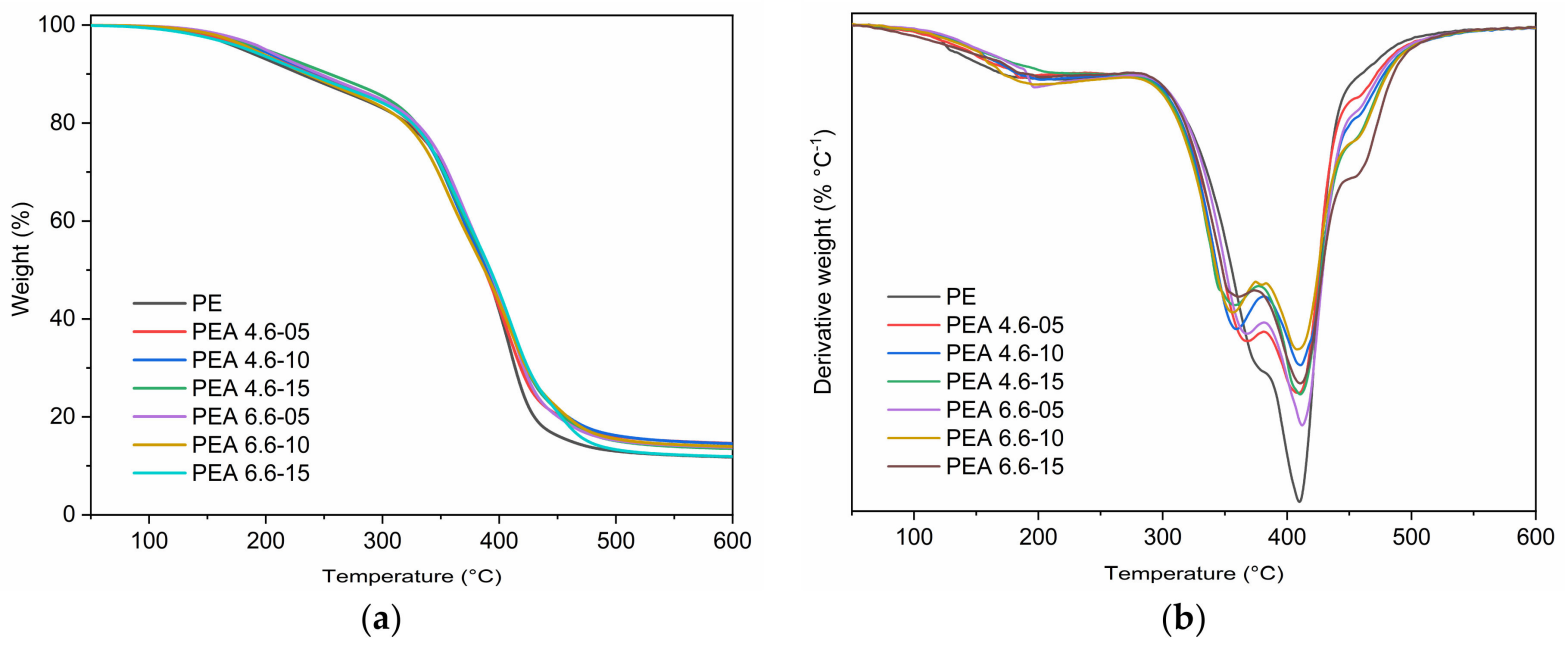
TGA thermograph (**a**) and dTG (**b**) of the cured resins.

The TGA results of the cured samples are given in Figure 8. The thermal degradation of the crosslinked resins occurs in three main steps as shown by the first derivative of the of the TGA curve (dTG graph). In the first step, a mass loss of 10%–15% is observed by 250 °C, which is assumed to correspond to non-reacted oligomers that have not been incorporated to the crosslinked network. However, the introduction of an amido diol seems to enhance the thermal stability of the material, since an increase of 5 to 16°C was found in the T_d,10%_ data that are shown in Table 5. In the second step of degradation, polyester segments decompose in the range of 350–400 °C, which is very common among polyester resins [55,56]. The incorporation of the amido diol leads to an extra peak in the dTG graph, which originally appeared as a shoulder peak for resin PE and as the amido diol mol% increases, that peak gets separated from the main polyester degradation peak and two clear peaks can be observed around 350 °C and 400 °C. Although it is unclear why this splitting occurs, it might be associated with the fact that the crosslinking density becomes sparser by introducing higher amido diol mol% in the polymer chains. In the third and final step, the amide segments are degrading, as it is shown by the third peak of the dTG graph, around 450 °C, the intensity of which increased along with the amido diol content of the resins. Lastly, the resins containing amide moieties presented higher residues at 800 °C than the PE resin, while resins containing amido diol 4.6 present slightly higher values for both T_d,10%_ and R_800°C_, suggesting better thermal stability.

## 4. Conclusions

In this work, we report the synthesis of bio-based unsaturated poly(ester amide)s, trying to overcome aza-Michael additions and isomerization reactions that usually take place when itaconic acid and primary diamines are reacted under polycondensation conditions. Our synthetic strategy involved the use of preformed symmetrical amido diols with two internal amide bonds. Evaluation of structural composition verified the successful synthesis of the desired poly(ester amide)s. In addition, both undesired side reactions were effectively suppressed even at amido diol contents of up to 15 mol%. The introduction of amide bonds in the polymer structure had a huge impact on the physicochemical properties of the resins e.g., the viscosity and reactivity toward UV-induced crosslinking. The latter was affected, as the conversion of the double bonds along with the rate of polymerization were lowered at higher amido diol content. However, at lower amounts of the amido diol building blocks incorporated into the PEA resins, the curing behavior was not significantly affected. For PEA with 10 mol% of the amido diol 4.6, the conversion and ROP was even higher than for the neat PE. In addition, all PEA resins exhibited an intriguing thixotropic behavior. This feature is very interesting, and it could be achieved even with amido diol loadings as low as 5 mol%. Furthermore, despite the lower conversion of some PEA resins, all samples were still suitable for UV-curing applications. The cured test specimen obtained in this study were examined on their thermomechanical properties by means of DMA, with a considerable impact of the amido diol on the properties of the samples. Therefore, the PEAs described in this study could be suitable as additives for certain UV-curing application where thixotropic behavior of the resins is desired.

## Figures and Tables

**Table 1:** Composition of the unsaturated neat polyester and poly(ester amide) resins synthesized. DMI: dimethyl itaconate, DMS: dimethyl sebacate.

Sample	DMI (eq)	DMS (eq)	1,3 PD (eq)	2,3 BD (eq)	Is (eq)	Gly (eq)	Amido Diol (eq)	Amido Diol (mol%)
PE	0.8	0.2	0.75	0.25	0.125	0.125	-	-
PEA 4.6-05	0.8	0.2	0.6875	0.25	0.125	0.125	0.0625	5
PEA 4.6-10	0.8	0.2	0.625	0.25	0.125	0.125	0.125	10
PEA 4.6-15	0.8	0.2	0.5625	0.25	0.125	0.125	0.1875	15
PEA 6.6-05	0.8	0.2	0.688	0.25	0.125	0.125	0.0625	5
PEA 6.6-10	0.8	0.2	0.625	0.25	0.125	0.125	0.125	10
PEA 6.6-15	0.8	0.2	0.5625	0.25	0.125	0.125	0.1875	15

**Table 2:** Viscosity values of the polymers at different shear rates and percentage of the initial viscosity recovered at the end of the experiment.

Sample	Starting Viscosity (Pa·s at 1 s^−1^)	Viscosity at High Shear Rate (Pa·s at 100 s^−1^)	% of Starting Viscosity at High Shear Rate	Viscosity at Recovery (Pa·s at 1 s^−1^)	% of Recovered Viscosity
PE	0.84	0.84	-	0.84	-
PEA 4.6-05	3.1	2.75	88.7	2.83	91
PEA 4.6-10	181	7	3.8	123	69
PEA 4.6-15	1127	30	2.6	773	68
PEA 6.6-05	22.9	3.37	14.7	22.3	97
PEA 6.6-10	258	10	3.8	251	95
PEA 6.6-15	Unable to measure at 25 °C				

**Table 3:** Double-bond density (DBD), theoretical and experimental enthalpy of curing, conversion, and maximum rate of polymerization (ROP) of the resins at 25 °C.

Sample	DBD (mmol/g)	ΔH_theo_ (J/g)	ΔH (J/g)	C (%)	ROP (1000 s^−1^)
PE	3.7	224.5	127	57	8.6
PEA 4.6-05	3.4	206.3	111	54	10.6
PEA 4.6-10	3.2	194.1	112	58	10.8
PEA 4.6-15	3.0	182.0	85	47	6.4
PEA 6.6-05	3.4	206.3	113	55	9.5
PEA 6.6-10	3.2	194.1	92	48	7.7
PEA 6.6-15	3.0	182.0	83.	46	6.3

**Table 4:** DMA results of the cured resins.

Sample	E’ at 25 °C (GPa)	E’ at 115 °C (GPa)	Crosslink Density (10^6^ × mol × m^−3^)	Tan δ Peaks (°C)	
PE	11.74	1.58	0.163	-	71
PEA 4.6-05	11.02	1.19	0.123	−0.4	69
PEA 4.6-10	5.41	0.51	0.053	3.6	63
PEA 4.6-15	2.06	0.22	0.023	6.8	55
PEA 6.6-05	6.70	0.63	0.065	−9	68
PEA 6.6-10	3.01	0.28	0.029	4	61
PEA 6.6-15	2.03	0.14	0.0145	17	58

**Table 5:** TGA results of the cured resins.

Sample	T_d,10%_ (°C)	R_800 °C_ (%)
PE	230	11.2
PEA 4.6-05	242	13.3
PEA 4.6-10	245	13.8
PEA 4.6-15	255	12.8
PEA 6.6-05	246	13.1
PEA 6.6-10	235	13.3
PEA 6.6-15	237	11.3

## Supplementary Materials

The following are available online at https://www.mdpi.com/2073-4360/12/4/980/s1, Figure S1: ^1^H NMR spectra of the resins containing amido diol 6.6., Figure S2: Close up from ^1^H NMR spectra. Top row AD 4.6, bottom row AD 6.6.
