# Supplementary material for: Straightforward Synthetic Protocol to Bio-Based Unsaturated Poly(ester amide)s from Itaconic Acid with Thixotropic Behavior
Source: Polymers (Basel). 2020 Apr 22;12(4):980. doi: 10.3390/polym12040980 (PMC7240367; doi:10.3390/polym12040980)
Supplement: Supplementary file 1 [file polymers-12-00980-s001.pdf]

polymers

Straightforward Synthetic Protocol to Bio-Based Unsaturated Poly(ester amide)s from Itaconic Acid with Thixotropic Behavior

Lazaros Papadopoulos [1], Marcel Kluge [2,3], Dimitrios N. Bikiaris [1] and Tobias Robert [2,*]

1. Laboratory of Polymer Chemistry and Technology, Department of Chemistry, Aristotle University of Thessaloniki, GR-541 24 Thessaloniki, Greece; lazaros.geo.papadopoulos@gmail.com (L.P.); dbic@chem.auth.gr (D.N.B.)
2. Fraunhofer Institute for Wood Research – Wilhelm-Klauditz-Institut WKI, Bienroder Weg 54E, 38108 Braunschweig, Germany; marcel.kluge@wki.fraunhofer.de
3. Institute for Chemistry of Renewable Resources, Department of Chemistry, University of Natural Resources and Life Sciences Vienna, Konrad-Lorenz-Straße 24, 3430 Tulln, Austria

* Correspondence: tobias.robert@wki.fraunhofer.de; Tel.: +49-531-2155-357 (T.R.)

Received: 30 March 2020; Accepted: 19 April 2020; Published: date

Supporting Information

Detailed analysis of ^{1}H NMR spectra obtained for synthesized polymers

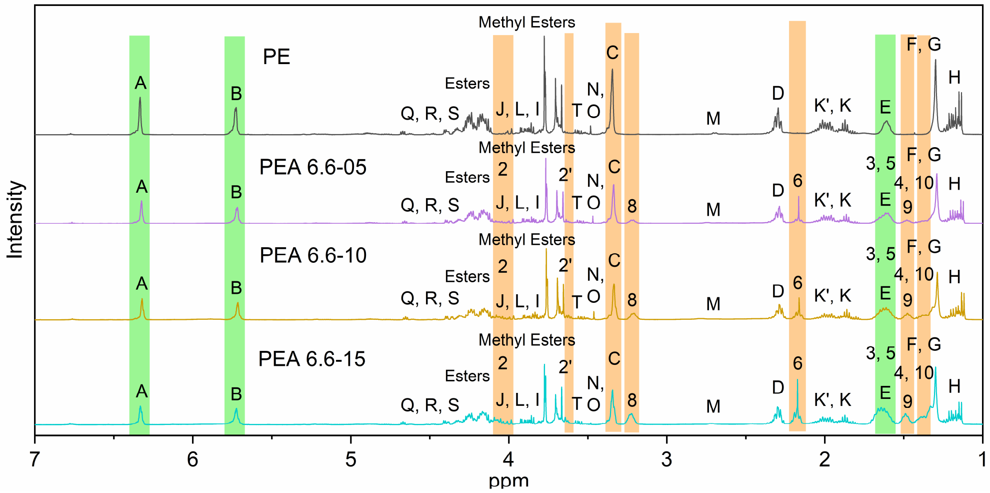

Figure S1. ^{1}**H** NMR spectra of the resins containing amido diol 6.6.

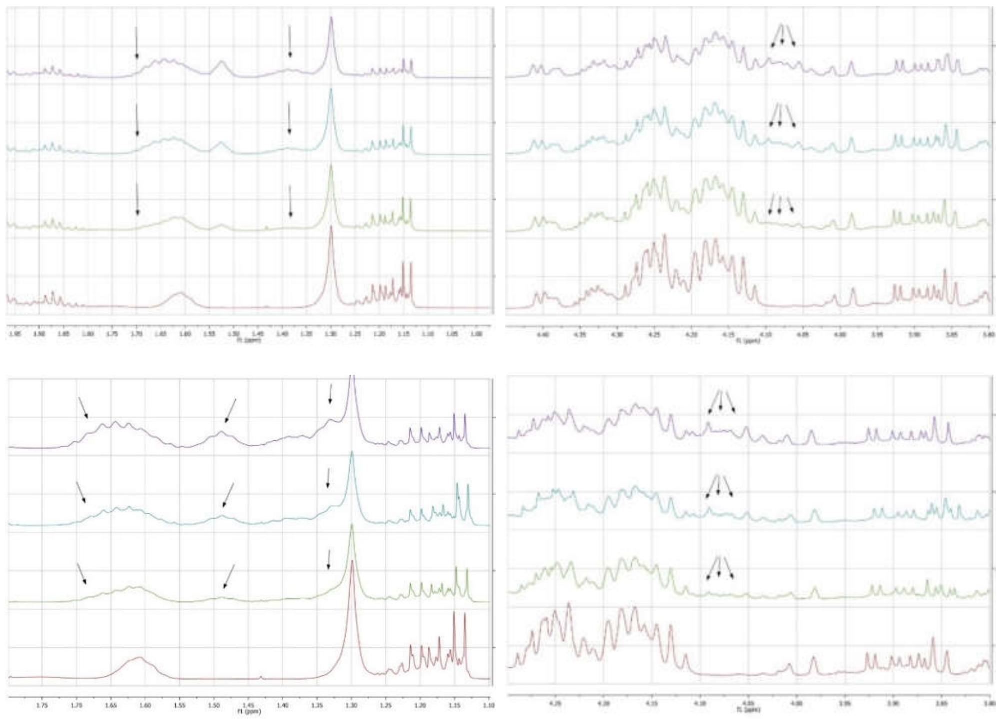

Figure S2. Close up from ^{1}H NMR spectra. Top row AD 4.6, bottom row AD 6.6.

From the structure of itaconic acid three peaks can found in Figure S1 namely "A", "B" and "C". Proton marked as "A" is more deprotected than the proton marked as "B" as it is located closer to the ester bond while protons of the methyl group are also visible at 3.35 ppm, marked as "C". In reactions where itaconic acid is involved a common phenomenon is its isomerization to mesaconic acid especially when catalytic amounts of a base are present at elevated temperatures [1]. When the isomerization occurs, the double bond peak shifts to 6.7 ppm while a singlet appears around 2.3 ppm. In the spectra of the neat polyester, PE, the ratio between the peaks of itaconic and mesaconic acid is 1:0.01 so the isomerization took place to very limited extend. Proceeding to the peaks from the structure of sebacic acid, the protons near the carbonyl group are the most deprotected and they appear at 2.3 ppm marked as "D". The protons "E" from the methyl groups near the "D" position appear at 1.65 ppm and finally, protons "F" and "G" appear at 1.3 ppm.

The second section of ^{1}H NMR analysis concerns the peaks derived from the diols used for the synthesis of the resins. Beside the peaks of the main structure of the diols, some free -OH groups are to be expected, as a 0.3 excess was used during the synthesis. As far as the esterified -OH groups are concerned, the peaks of the protons near the ester groups appear between 4.1 and 4.3 ppm, but as they overlap, we were unable to assign each one to the respective ester. So, protons marked as "H", "I" and "J" on the structure of 2,3-butanediol appear at 1.15 ppm, 3.8 ppm and 3.98 ppm respectively. 1,3-Propanediol protons "K" appear at 1.85 ppm when they are mono-esterified and they shift to the left at 2.05 ppm when both hydroxyl groups are esterified, "L" and "M" protons appear at 3.77 ppm and 2.70 ppm and glycerol protons "N", "O" and "P" at 3.42 ppm, 3.38 ppm and 4.48 ppm respectively. Lastly, the isosorbide structure provides the "Q" protons at 4.4 ppm, "R" protons at 4.25 ppm, "S" protons at 4.15 ppm and "T" protons at 3.65 ppm. The above-mentioned results verify the successful incorporation of all diols into the structure of the resins. Since an excess of 0.3 was used for the synthesis, the presence of -OH groups can be observed. However, they originate from monoesterified diols, the end groups of the polymer chains. They are shifted compared with the peaks of the spectra of the unreacted monomers, thus proving that there is not a significant amount of unreacted monomers present in the final resins.

For the resins containing the amido diols, the peak assignment is the same as it was described above for the PE resin, with the new peaks emerging belonging to the protons of the respective amido

diols (highlighted with light orange in Figure S1). More specifically, for the resins containing the amido diol 4.6 the ester peaks are observed at 4.05 ppm as shown in the close-up Figure S2, and the protons near free -OH groups marked as "2" at 3.65ppm. The peak of the protons near nitrogen atoms marked as "8" appear at 3.26 ppm while the protons near the carbonyl group marked as "6" appear at 2.18 ppm. Finally, protons marked "3","4","5" and "9" appear between 1.35 and 1.7 ppm. For amido diol 6.6 we observe an additional peak at 1.3 ppm, corresponding to the protons marked as "10".

References

1. Farmer, T. J.; Clark, J. H.; Macquarrie, D. J.; Ogunjobi, J. K.; Castle, R. L. Post-polymerisation modification of bio-derived unsaturated polyester resins via Michael additions of 1,3-dicarbonyls. *Polym. Chem.* **2016**, *7*, 1650–1658, doi:10.1039/c5py01729g.
